# Local governance dynamics in the Colombian health system: an exploratory cross-sectional study among public health sector officials

**DOI:** 10.1057/s41271-026-00635-8

**Published:** 2026-06-01

**Authors:** Natalia Losada-Trujillo, Javier Eslava-Schmalbach, Carol C. Guarnizo-Herreño

**Affiliations:** 1 School of Medicine, Universidad Nacional de Colombia, Bogotá, Colombia; 2 Hospital Universitario Nacional de Colombia, Bogotá, Colombia; 3 School of Dentistry, Universidad Nacional de Colombia, Bogotá, Colombia

**Keywords:** Health system governance, Financing fragmentation, Health policy, Colombia, Decision-making, Public health officials

## Abstract

Health system governance is essential for effective and equitable healthcare delivery. In Colombia, persistent challenges, including delays, denials, barriers to care, and poor system coordination, suggest weaknesses in local governance. This study aimed to explore local health system governance in Colombia by assessing institutional practices, decision-making processes, and perceptions of public health sector officials. Exploratory cross-sectional study using self-administered online surveys (May–July 2025) with a convenient non-probabilistic sample of 44 health sector officials in stewardship, planning, and oversight roles. Descriptive analyses were performed. While we identified high institutional planning capacity, respondents reported limited decision-making autonomy, external pressures on planning and budget allocation, poor integration of information systems, perceived insufficiency of the benefits package and capitation payment, irregular provider payments, and tariff variability. Findings suggest weak local health system governance in Colombia, highlighting the need to implement a comprehensive reform including strengthened stewardship, information systems integration, adequate financing, and fair provider payments.

**Supplementary Information:**

The online version contains supplementary material available at 10.1057/s41271-026-00635-8.

## Key messages

- Survey findings from public health officials suggest weak local governance in the Colombian health system, characterized by external pressures on decision-making, fragmented information systems, insufficient funding, and a lack of national regulation of payment methods.
- Public health officials report pressure from external interest holders influencing planning processes and budget allocation in the health sector.
- Weak local governance in the Colombian health system may contribute to longer waiting times, increased administrative burden, and higher out-of-pocket expenses for patients.

## Introduction

Health system governance refers to the processes, structures, and institutions responsible for directing and coordinating the functioning of a country’s health system [1, 2]. It encompasses the management of relationships among system actors, decision-making, and policy implementation, to ensure equitable, sustainable, and high-quality healthcare [1–3].

According to the 2023 Health System Performance Assessment (HSPA) framework of the World Health Organization and the European Observatory on Health Systems and Policies, the subfunctions of governance encompass: policy and vision (system planning and evaluation); multisectoral collaboration (coordination between the health sector and other sectors); population and civil society participation (stakeholder involvement in decision-making); information and knowledge (strength of the information system and evidence-informed decision-making); and legislation and regulation (regulatory oversight and safeguards against undue interest group influence) [4]. The study of health systems governance is particularly relevant, as governance deficiencies can lead to health policy failure, driven by corruption, incompetence, nepotism, unintended consequences of poorly conceived policies, and limitations in long-term planning [1]. These factors impede progress toward universal health coverage (UHC), deepen inequities in access and quality of care, and ultimately contribute to adverse health outcomes [5].

Colombia presents a particularly relevant case for examining these governance dynamics, as its health system combines a complex institutional architecture with persistent challenges in effective access to services [6]. Established under the Law 100 of 1993, the system created the General System of Social Security in Health (SGSSS, from the Spanish equivalent) and allowed the creation of insurance entities (*Entidades Promotoras de Salud, EPS*) that manage risk through a capitation premium (*Unidad de Pago por Capitación, UPC*) and administer the provision of services through health service providers (*Instituciones Prestadoras de Servicios de Salud, IPS*) [7].

The system operates under three affiliation regimes that are meant to guarantee access to health services for the entire population, regardless of ability to pay [7], achieving an insurance coverage of 98.4% in 2025 [8]. It operates under a managed competition model, in which insurers compete for user enrollment, and users may choose their healthcare provider from among those offered by their insurer, with a standardized benefits package, within a state-regulated framework [9, 10]. From a financing perspective, and following the HSPA framework [4], the system exhibits low fragmentation in revenue collection and risk pooling, as it draws on multiple funding sources centralized through an entity (ADRES) [11]; in contrast, its purchasing function is highly fragmented, given that multiple insurers independently contract with heterogeneous provider networks under diverse payment arrangements [12].

The system features a decentralized administrative structure, with health secretariats at the local level (departments and municipalities) and the Ministry of Health and Social Protection at the national level providing direction and oversight within a framework established by national legislation [7, 13]. It also has the inspection, surveillance, and control of the National Superintendency of Health to protect the rights of members, and entities that oversee fiscal management, such as the Comptroller General of the Republic and the Attorney General of the Nation [7, 13].

In 2008, a severe crisis in the Colombian health system was formally recognized, primarily evidenced by the citizens’ massive utilization of legal services in response to the denial, delay, and postponement of procedures and medications, largely driven by implicit rationing by insurers and providers [6, 14]. Additional factors contributing to this crisis include corruption involving multiple system actors, which, based on cases reported in the media between 2016 and 2022, resulted in estimated losses of approximately 411.1 million USD [15], as well as the country’s internal armed conflict, which in certain regions has contributed to the diversion of health resources and to the control of health service provision and financial flows by armed groups [16].

Understanding local governance processes within the Colombian health system is therefore essential for future reforms aimed at advancing the system toward UHC and equity in health care [1, 2]. This study aims to explore governance dynamics at the local level through the assessment of institutional practices, decision-making processes, and perceptions of public health sector officials in Colombia.

Findings of this study may contribute to understand the local governance dynamics within the Colombian health system and offer insights applicable to countries with similar characteristics. Furthermore, they may help identify factors that undermine governance performance and contribute to health policy failures, ultimately hindering equitable access to health services, exacerbating inequities in access and quality of care, impeding progress toward UHC, and contributing to adverse health outcomes.

## Data and Methods

In this descriptive exploratory cross-sectional study, we focused on responses from active public health sector officials with decision-making functions in stewardship, planning, and oversight of the Colombian health system. We applied a convenience non-probabilistic convenience sampling design (with no a priori sample size calculation), seeking to include officials from all regions of Colombia.

First, we designed a survey to capture information related to sociodemographic characteristics, institutional practices, decision-making dynamics, and perceptions of the health system directed at public officials who are decision-makers in the health system. The survey underwent pilot testing with subsequent adjustments to wording and response options. The final version was stored in REDCap [17] (Supplementary Material, Part 1).

Next, we identified health secretaries, undersecretaries, directors, deputy directors, and managers of public health areas in different Colombian departments, municipalities, and capital cities, and sent 98 invitations to complete the online survey. We described the study and requested to disseminate the survey among officials with decision-making functions and stewardship, planning, and oversight roles of the system in their territories. To encourage participation, we randomly assigned economic incentives.

Surveys were administered between May and July 2025; 44 (44.9%) surveys were completed with approved informed consent were used for the analysis.

A univariate descriptive analysis was conducted, presenting categorical variables as absolute and relative frequencies. The unit of analysis was the individual respondent, and only those meeting the predefined inclusion criteria were included in the analysis. Statistical analyses were performed using Stata version 15 [18].

### Ethics declaration

The project was approved by the ethics committee of the Faculty of Medicine of the National University of Colombia (B.FM.1.002-CE-133-24). The research complied with Colombian regulations and the Declaration of Helsinki. Informed consent was obtained from all participants.

## Results

The 44 participants included were grouped into three categories: territorial health secretaries (*n* = 17; 38.6%), managers (*n* = 20; 45.5%), and other officials with decision-making capacity in the public sector (*n* = 7; 15.9%). Of the participants, 79.6% (*n* = 35) were women, and the most frequent age group was 36 to 50 years (*n* = 26; 59.1%). In terms of educational level, specialists predominated (*n* = 24; 54.6%), followed by master’s degree holders (*n* = 12; 27.3%), and the majority of respondents had more than 6 years working in public health entities (*n* = 21; 47.7%) (Table 1).

**Table 1:** Sociodemographic characteristics of respondents by categories

Variable	Total (*n* = 44)	Territorial health secretaries (*n* = 17)	Health sector managers (*n* = 20)	Other positions (*n* = 7)
Sex, *n* (%)				
Female	35 (79.5)	13 (76.5)	16 (80.0)	6 (85.7)
Male	9 (20.5)	4 (23.5)	4 (20.0)	1 (14.3)
Age group, *n* (%)				
18–35 years	11 (25.0)	5 (29.4)	5 (25.0)	1 (14.3)
36–50 years	26 (59.1)	9 (52.9)	12 (60.0)	5 (71.4)
51–70 years	7 (15.9)	3 (17.6)	3 (15.0)	1 (14.3)
Highest academic degree attained, *n* (%)				
High school	1 (2.3)	0 (0.0)	1 (5.0)	0 (0.0)
Undergraduate	7 (15.9)	1 (5.9)	3 (15.0)	3 (42.9)
Specialist	24 (54.6)	13 (76.5)	9 (45.0)	2 (28.6)
Master’s degree	12 (27.3)	3 (17.6)	7 (35.0)	2 (28.6)
Years working in public health institutions, *n* (%)				
< 1 year	3 (6.8)	0 (0.0)	1 (5.0)	2 (28.6)
1–3 years	14 (31.8)	7 (41.2)	6 (30.0)	1 (14.3)
4–6 years	6 (13.6)	3 (17.7)	2 (10.0)	1 (14.3)
> 6 years	21 (47.7)	7 (41.2)	11 (55.0)	3 (42.9)
Municipal population size, *n* (%)				
≥ 500,001	13 (29.6)	2 (11.8)	11 (55.0)	0 (0.0)
50,001–500,000	10 (22.7)	3 (17.6)	1 (5.0)	6 (85.7)
20,001–50,000	10 (22.7)	6 (35.3)	4 (20.0)	0 (0.0)
≤ 20,000	11 (25.0)	6 (35.3)	4 (20.0)	1 (14.3)

*n* = number of participants; % = percentage within each category

Regarding regional distribution, the greatest participation came from Bogotá and Boyacá (*n* = 6; 13.6% each), followed by Cundinamarca (*n* = 5; 11.4%) and Antioquia (*n* = 4; 9.1%) (Fig. S1). Most health secretaries worked in municipalities of 50,000 inhabitants or fewer (*n* = 12; 70.6%), managers in municipalities of 500,001 inhabitants or more (*n* = 11; 55.0%), and other officials in municipalities of 50,001–500,000 inhabitants (*n* = 6; 85.7%) (Table 1).

**Fig. 1:**
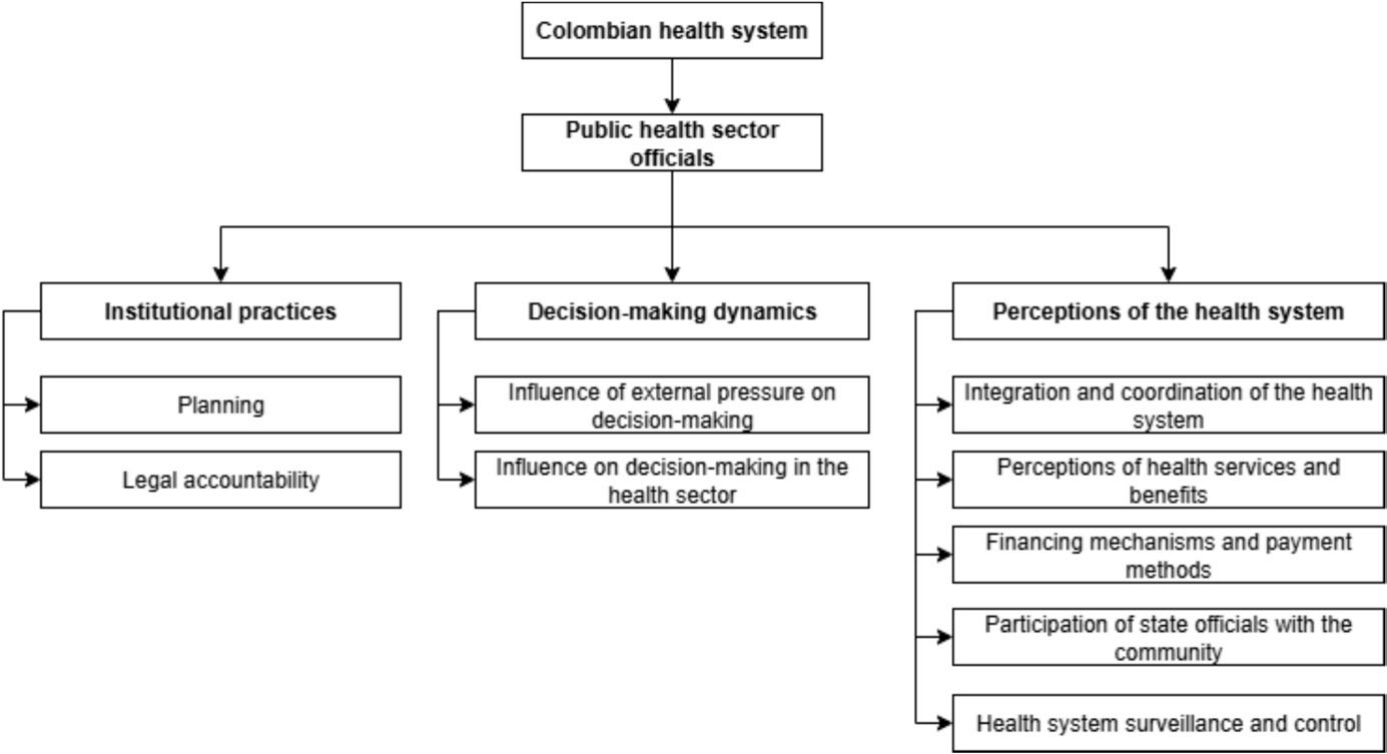
Institutional practices, decision-making dynamics, and perceptions of public health sector officials

The information collected was organized into three analytical dimensions: institutional practices, decision-making dynamics, and perceptions about the health system, each with specific subcategories. A framework that emerged from the data analysis process was used to structure the presentation of findings (Fig. 1).

Institutional practices were grouped into two dimensions: planning and legal accountability (Supplementary Material, Tables S1 and S2).

The majority of respondents reported that their institution had periodic plans to address health problems in their territory (*n* = 43; 97.7%). Likewise, their level of participation in planning exercises was high in most cases (*n* = 39; 90.7%), and nearly all reported that they use information about population health to identify priority areas in developing their entity’s strategic improvement plans (*n* = 41; 95.5%) (Table S1).

The frequency with which respondents reported awareness of “*tutela”* actions (a constitutional mechanism through which citizens can claim the protection of their fundamental rights before a judge in Colombia [13]) filed against their respective institutions for alleged violations of the right to health was predominantly high (*n* = 19; 43.2%) and occasional (*n* = 15; 34.1%) (Table S2).

The decision-making dynamics of public sector health officials were grouped into two dimensions: influence of external pressure: pressure from external groups or coalitions that affected decisions; and influence on officials’ decision-making: actors’ own responsibilities and competencies (Supplementary Material, Tables S3 and S4).

Of the respondents, 56.8% (*n* = 25) considered that officials from some public entities could feel pressured by external groups to modify health system planning; among these, the majority considered that these pressures could increase patient care and problem resolution times (*n* = 18; 72.0%) and increase administrative processes for patients (*n* = 14; 56.0%) (Table S3).

Regarding the perception of pressure from external groups or coalitions on public officials to make changes in financing mechanisms and budget allocation, 34.1% of respondents considered that officials could be under pressure (*n* = 15). Among these, the majority considered that this could increase administrative processes (*n* = 11; 73.3%), waiting times for care and problem resolution, out-of-pocket expenses, and medical consultations and laboratory tests for patients (*n* = 8; 53.3% each) (Table S3).

**Fig. 2:**
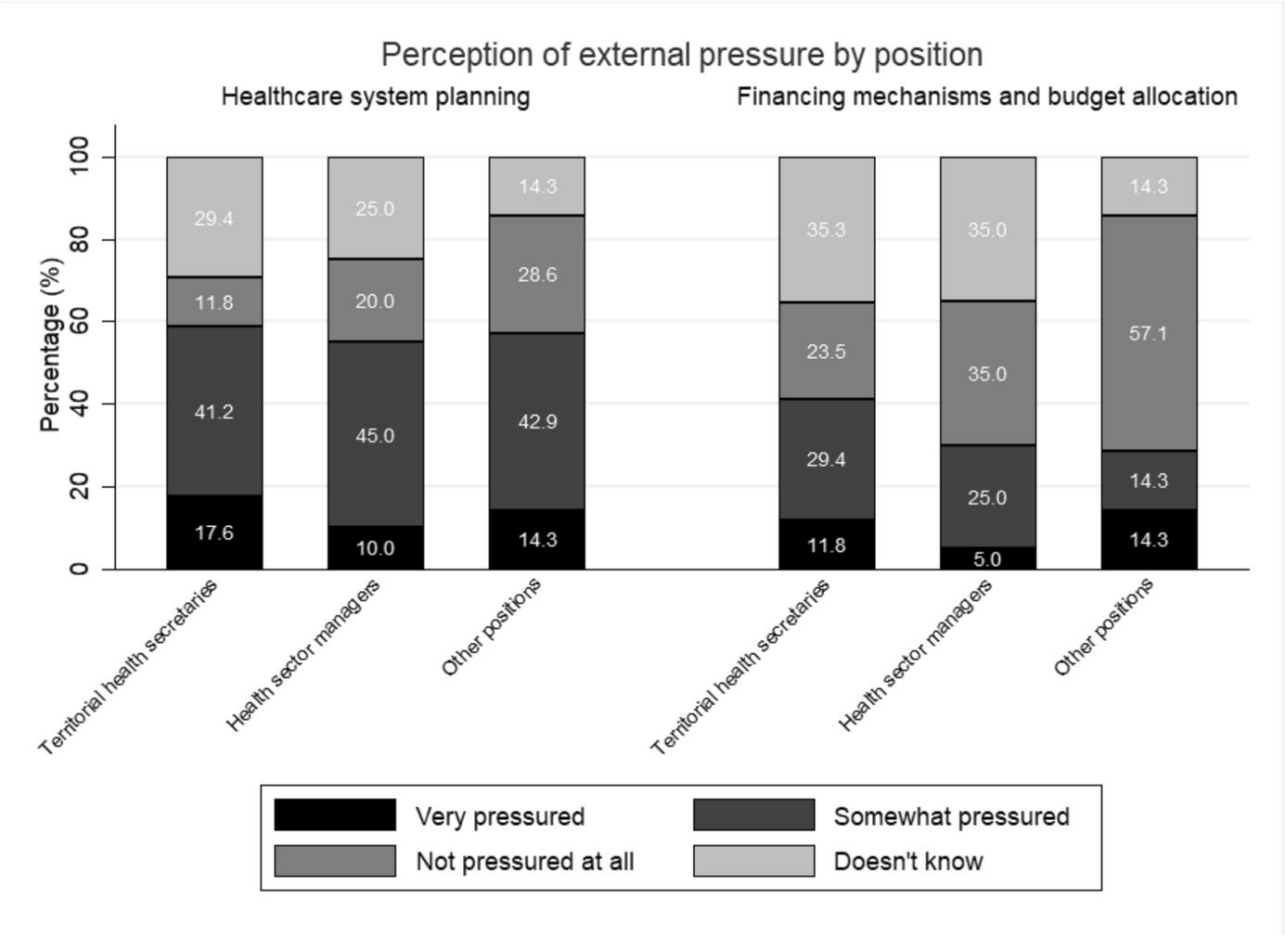
Perception of external pressure on decision-making by position in healthcare system planning and financing mechanisms and budget allocation

Figure 2 shows the distribution of perception of external pressure by groups or coalitions according to the respondent’s position for both components evaluated: health system planning and financing mechanisms and budget allocation.

The majority of respondents considered that they required greater decision-making power over the operation of public hospitals and insurance programs in their territory (*n* = 34; 77.3%). Territorial health secretaries and managers reported having the capacity to successfully suggest the enrollment of new beneficiaries in programs (*n* = 9; 53% and *n* = 9; 45%, respectively); while officials in other positions reported not having suggested such actions (*n* = 4; 57.1%) (Table S4).

Respondents’ perceptions regarding the health system were grouped into five dimensions: integration and coordination of the health system, health services and benefits, financing mechanisms and payment methods, participation of state officials with the community, and health system surveillance and control (Supplementary Material, Tables S5-S9).

The majority of respondents considered that the governing institutions of the health system were moderately integrated (*n* = 18; 41.0%) or integrated (*n* = 12; 31.8%) with health service provider institutions. Regarding public and private sector information systems, most respondents considered that they were either highly or somewhat integrated (*n* = 24; 54.5%), while the remaining half considered that they were not integrated at all (*n* = 20; 45.5%). Respondents identified the main consequences of lack of integrated information among providers, payers, and patients as: increased care and problem resolution times (*n* = 34; 77.3%), increased administrative procedures (*n* = 31; 70.5%), increased out-of-pocket expenses (*n* = 25; 56.8%), and increased adverse health outcomes (*n* = 21; 47.7%) (Table S5).

The majority considered that the UPC was insufficient for health service management and administrative costs in Colombia (*n* = 36; 81.8%). A minority of respondents considered that, given the population’s needs, the services and procedures included in the benefits package were sufficient (*n* = 12; 27.3%) (Table S6).

The majority of respondents considered that health service providers were paid on time only sometimes (*n* = 24; 54.6%), followed by never (*n* = 10; 22.7%) and almost always (*n* = 8; 18.2%). The majority considered that differential rates existed among health service providers for the same services (*n* = 41; 93.2%) (Table S7).

The majority of respondents considered that the participation of state officials with the community was partially satisfactory (*n* = 26; 59.1%) or satisfactory (*n* = 14; 31.8%) (Table S8).

Respondents considered that the role in oversight and control of the health system was somewhat effective for the Superintendency (*n* = 23; 52.3%), the Comptroller’s Office (*n* = 22; 50%), and the Attorney General’s Office (*n* = 24; 54.6%) (Table S9).

## Discussion

We explored local health system governance in Colombia through the assessment of institutional practices, decision-making dynamics, and perceptions of public health sector officials. We identified high institutional planning capacity, with significant participation of officials in planning exercises. This finding is consistent with what is established in Colombian laws on health system organization [7, 19] and with what was reported in the survey conducted by the Organization for Economic Co-operation and Development (OECD) and the Inter-American Development Bank (IDB) in 2018 [12], which confirm that Colombia has national strategic plans, legislation to guarantee quality of care, and a clear organizational structure, where coverage decisions and public health policy design are based on evaluations of scientific evidence [12].

Regarding decision-making dynamics, the results revealed that public health sector officials experience varying degrees of pressure from external groups to modify institutional planning exercises and budget allocation, generating increased waiting times, greater administrative burden, and increased out-of-pocket expenses for patients. This phenomenon may be explained by several interconnected factors. First, it is consistent with a qualitative study on health decision-making dynamics in Colombia, which identified that the system’s market mechanisms and competition generate pressure on institutional insurance and service provision processes, where provider institutions are threatened with closure in the face of insures delays or lack of payments, and different system reforms, prioritizing market laws over citizens’ rights [20]. Additionally, this external pressure may reflect the influence of political actors on the system [21] and documented cases of corruption within it [15]; in this regard, a qualitative study with 141 health system actors found that 72% identified corruption as the system’s main problem [22]. The effects of the armed conflict on institutional decision-making in certain regions may also contribute to this dynamic [16].

Regarding perceptions about the health system, respondents identified limited or nonexistent integration between public and private information systems. This finding was consistent with previous studies [23, 24] that demonstrated that medical records information is not effectively shared among providers, affecting system efficiency, quality of services, and coordination between levels of care.

Likewise, findings of this study revealed the perception of inadequacy in the services and procedures of the benefits package, as well as in the UPC. This perception was consistent with a study that calculated the loss ratio of the UPC for the years 2017 to 2021 in all insurers and identified insufficient resources for health insurance operations [25]. In contrast, according to the Ministry of Health, in 2024, 80% of legal actions (*tutelas*) filed for failure to comply with the right to health were related to services financed/covered by the Benefits Plan, with the main causes being postponement and delay in provision [6].

Similarly, respondents perceived irregularities in financing mechanisms and payment methods, evidenced by variations in payment times to providers and rate differences for the same services. These findings are in line with a qualitative study that demonstrated that this model of voluntary agreements could affect care coordination by incentivizing competition over adequate service provision [24].

The study’s strengths include the participation of officials from diverse roles and territorial levels, enabling the capture of multiple perspectives. However, findings should be interpreted with caution due to the exploratory nature of the study, non-representative sample, and potential selection bias related to the sampling strategy and economic incentive offered as a token of appreciation for the participants’ time. The anonymous design prevented the identification of the proportion of respondents recruited via snowball sampling. Only participants meeting the inclusion criteria were analyzed. Future research should examine the impact of weak governance on health outcomes and the mechanisms through which external pressures and corruption influence decision-making.

Taken together, the study findings along with what has been reported in the existing literature suggest the existence of weak health system governance, where there is external pressure in health decision-making, poor integration of information systems, financial insufficiency of the system, and lack of national rate regulation between insurers and providers. These findings support what was identified in a qualitative study that documented that health system leaders perceived low system governance, given by the presence of corruption, lack of coordination among actors, and underfunding of the system [22]. The findings suggest the need for structural reforms in the health system that guarantee effective control and stewardship, ensure adequate financing, prevent corruption, integrate information systems, and regulate rates at the national level.

## Conclusions

The Colombian health system exhibits weak local governance, characterized by external pressures on decision-making, financial insufficiency, the absence of rate regulation, and a lack of information system integration. Health systems in Colombia and in countries with similar contexts would benefit from promoting coordination and sustainability by strengthening stewardship and financial planning, integrating information systems, preventing corruption through enhanced monitoring, regulating payment methods and processes at the national level, and ensuring adequate financing to advance toward quality, equitable and universal health systems. Our findings highlight the need for a comprehensive health system reform in Colombia, including strengthened stewardship, information systems integration, adequate financing, and fair provider payments.

## Supplementary Information

Below is the link to the electronic supplementary material.

Supplementary file1 (DOCX 1763 KB)

## Data Availability

The data that support the findings of this study are not publicly available due to privacy and ethical considerations, as they contain sensitive information from anonymous respondents. Aggregated or anonymized data may be available from the corresponding author upon reasonable request, subject to institutional ethical approval and data protection regulations.
