# Supplementary material for: Local governance dynamics in the Colombian health system: an exploratory cross-sectional study among public health sector officials
Source: J Public Health Policy. 2026 Jun 1;47(2):279–90. doi: 10.1057/s41271-026-00635-8 (PMC13278946; doi:10.1057/s41271-026-00635-8)
Supplement: Supplementary file 1 — Supplementary file1 (DOCX 1763 KB) [file 41271_2026_635_MOESM1_ESM.docx]

**Supplementary material: Local governance dynamics in the Colombian health system: an exploratory cross-sectional study among public health sector officials**

[**Supplementary material 1. Survey applied 2**](#_gn8si69asns)

[**Figures 6**](#_uexesu2c96q8)

[**Figure S1. Distribution map of survey responses 6**](#_73a2b1m76hja)

[**Tables 7**](#_jib1qlk5li8q)

[**Table S1. Institutional practices of public health sector officials: Planning 7**](#_elkeiqm4vvzy)

[**Table S2. Institutional practices of public health sector officials: Legal accountability 7**](#_j3wipfs6tf90)

[**Table S3 Decision-making dynamics of public health sector officials: Influence of external pressure on decision-making 8**](#_fe1yfdot5eti)

[**Table S4. Decision-making dynamics of public health sector officials: Influence on decision-making in the health sector 10**](#_yre5sevk2uu0)

[**Table S5. Perceptions of public health sector officials regarding the health system: Integration and coordination of the health system 12**](#_t65x1mfjyh6d)

[**Table S6. Perceptions of public health sector officials regarding the health system: Perceptions of health services and benefits 13**](#_oapfwgtf9f6m)

[**Table S7. Perceptions of public health sector officials regarding the health system: Financing mechanisms and payment methods 14**](#_lzmklmbg65el)

[**Table S8. Perceptions of public health sector officials regarding the health system: Participation of state officials with the community 15**](#_j8ouci8we4md)

[**Table S9. Perceptions of public health sector officials regarding the health system: Health system surveillance and control 15**](#_g4vy1ta4sbof)

# **Supplementary material 1. Survey applied**

**Political and institutional determinants of policy reforms and other decision-making processes related to the fragmentation of health system financing in Colombia**

**What is your biological sex?**

Female O Male O

**What age range are you in?**

18–25 years O 26–30 years O 31–35 years O 36–40 years O 41–45 years O 46–50 years O 51–55 years O 56–60 years O 61–65 years O 66–70 years O Over 70 years O

**What is your current position?** ________________________________

**Department in which you hold office**: __________________________________

**Population of the municipality where you hold office:**

500,001 or more inhabitants O 100,001–500,000 inhabitants O 50,001–100,000 inhabitants O 30,001–50,000 inhabitants O 20,001–30,000 inhabitants O 10,001–20,000 inhabitants O 10,000 or fewer inhabitants O

**How long have you worked in public health sector entities?:**

Less than 1 year O 1–3 years O 4–6 years O More than 6 years O

**Highest level of academic training completed:**

Bachelor’s O Professional O Specialist O Master’s O Doctorate O Postdoctorate O

**Are there periodic plans related to major public health problems (annual, triennial, or quinquennial) in your institution?**

Yes O No O Does not know O

**To what extent have you personally participated in the planning exercises referred to in the previous question?**

A lot O Somewhat O Not at all O

**Do you use population health information to identify priority areas when developing strategic improvement plans in your institution?**

Yes O No O

**How would you describe the level of integration between governing health institutions and healthcare service providers?**

Highly integrated O Integrated O Moderately integrated O Poorly integrated O Not integrated O

**Given your population’s needs, do you believe the services and procedures included in the benefits plan are sufficient?**

Yes O No O

**Do you believe healthcare providers are paid on time?**

Always O Almost always O Sometimes O Never O

**Do you believe there are differential payment rates among healthcare providers for the same services?**

For no services O For some services O For all services O

**Do you believe that in some public entities, officials may feel pressured by external groups or coalitions to intervene in health sector planning?**

Highly pressured O Somewhat pressured O Not pressured O Do not know O

**As a result of such interventions, which of the following consequences could have occurred? (You may select more than one option, if applicable)**

Increased demand for services such as medical consultations and lab tests ☐

Increased administrative procedures for patients ☐

Longer waiting and problem-solving times for patients ☐

Higher out-of-pocket expenses for patients ☐

More negative health outcomes for patients ☐

Increased costs for healthcare providers ☐

Increased costs for payers ☐

None of the above ☐

**Do you believe that in some public entities officials may feel pressured by external groups or coalitions to suggest, propose, or oppose changes in financing and budget planning mechanisms within the health sector?**

Highly pressured O Somewhat pressured O Not pressured O Do not know O

**As a result of such changes, which of the following consequences could have occurred? (You may select more than one option, if applicable)**

Increased demand for services such as medical consultations and lab tests ☐

Increased costs for healthcare providers ☐

Increased costs for payers ☐

Increased administrative procedures for patients ☐

Longer waiting and problem-solving times for patients ☐

Higher out-of-pocket expenses for patients ☐

None of the above ☐

**How would you describe the participation of state officials in their interaction with the community regarding public health issues?**

Unsatisfactory O Partially satisfactory O Satisfactory O

**Do you believe that the Capitation Payment Unit (UPC) is sufficient to manage healthcare services and administrative costs in Colombia?**

Fully sufficient O Sufficient O Insufficient O Completely insufficient O

**What do you consider to be the consequences of the lack of integrated information among providers, payers, and patients? (You may select more than one option, if applicable)**

Increased diagnostic tests for patients ☐

Increased administrative procedures for patients ☐

Longer waiting and problem-solving times for patients ☐

Higher out-of-pocket expenses for patients ☐

More negative health outcomes for patients ☐

Increased costs for healthcare providers ☐

Increased costs for payers ☐

None of the above ☐

**Do you believe that public and private health information systems are integrated for healthcare service provision?**

A lot O Somewhat O Not at all O

**Do you believe you should have greater decision-making power over the management of public hospitals and insurance programs in your area/locality/municipality/department?**

Yes O No O

**How would you describe national government guidelines or regulations regarding how health budgets should be spent?**

It is not possible to make adjustments between spending categories O

In some cases, it is possible to make adjustments between spending categories O

It is always possible to make adjustments between spending categories O

Other O

**In the past 12 months, please indicate in which of the following areas you have made decisions regarding financial allocations or expenditures (You may select more than one option, if applicable):**

Enrollment of new beneficiaries ☐

Payment to existing healthcare providers ☐

Hiring of new providers ☐

Essential capital expenditure (infrastructure, technology) ☐

Flexible/discretionary spending ☐

None of the above ☐

Other ☐

**In your routine service delivery activities, if you disagree with the directives/orders of national-level health agencies, authorities, or ministries, what do you usually do?**

I can only implement what is required O

I contact the district administrator/mayor/head who may escalate the issue O

I write to regional/national project directors/coordinators requesting approval for changes O

Sometimes I ignore their orders O

Sometimes I only implement part of what is required O

I add my own priorities to the required activities O

**Which of the following initiatives have you proposed or implemented successfully?**

| **Initiatives** | **Suggested and successful** | **Suggested but unsuccessful** | **Not suggested** |
| --- | --- | --- | --- |
| Registering new beneficiaries in programs |  |  |  |
| Opening new clinics or medical offices |  |  |  |
| Extending staff/facility working hours |  |  |  |
| Introducing/improving complaint and claim resolution mechanisms |  |  |  |
| Introducing new services (included in benefits packages) |  |  |  |

**How effective do you consider the role of the National Health Superintendence in monitoring and controlling the health system in Colombia?**

Very effective O Somewhat effective O Not effective O Do not know O

**How effective do you consider the role of the Comptroller’s Office in monitoring and controlling the health system in Colombia?**

Very effective O Somewhat effective O Not effective O Do not know O

**How effective do you consider the role of the Attorney General’s Office in monitoring and controlling the health system in Colombia?**

Very effective O Somewhat effective O Not effective O Do not know O

**How often have you been aware of legal actions (tutelas)^1^ filed against your institution for failure to uphold the right to health?**

Very frequently O Sometimes O Never O Do not know O

^1^ The tutela is a mechanism for protecting citizens' constitutional rights through judicial authorities when those rights are violated or threatened by the action or omission of public authorities [(1)](https://www.zotero.org/google-docs/?tZkdfh).

# **Figures**

## **Figure S1. Distribution map of survey responses**


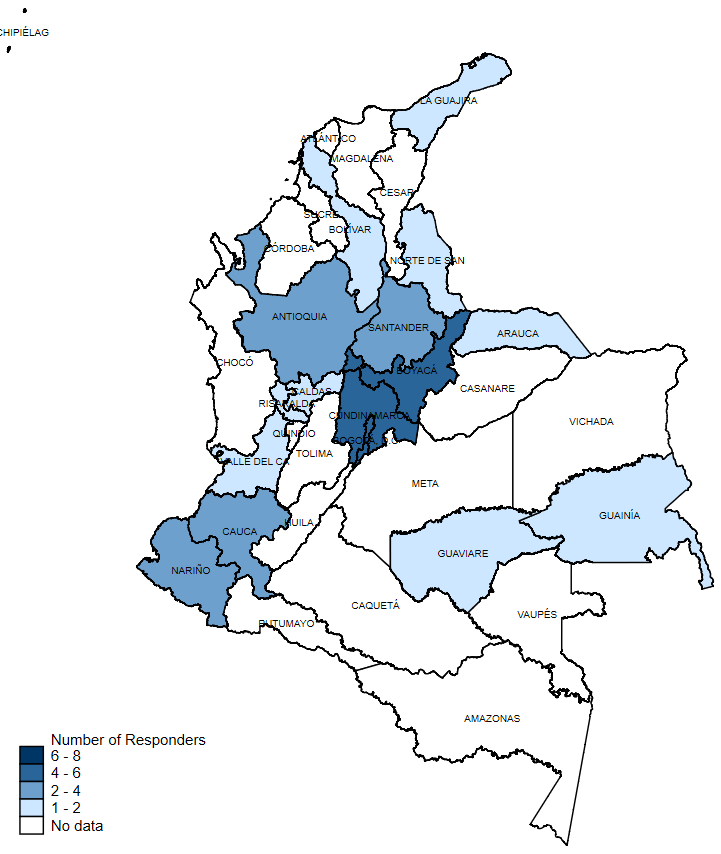


# **Tables**

## **Table S1. Institutional practices of public health sector officials: Planning**

| **Variable** | **Total (n=44)** | **Territorial health secretaries (n=17)** | **Health sector managers (n=20)** | **Other positions (n=7)** |
| --- | --- | --- | --- | --- |
| **Existence of periodic plans related to the most impactful health problems in the entity, n (%)** | 43 (97.7) | 17 (100.0) | 20 (100.0) | 6 (85.7) |
| **Level of personal participation in the planning exercises, n (%)** | | | | |
| A lot | 39 (90.7) | 15 (88.2) | 19 (95.0) | 5 (83.3) |
| Somewhat | 4 (9.3) | 2 (11.8) | 1 (5.0) | 1 (16.7) |
| **Use of population health information to identify priority areas during the development of strategic improvement plans in the entity, n (%)** | 42 (95.5) | 16 (94.1) | 19 (95.0) | 7 (100.0) |
| Note: n = number of participants; % = percentage within each category. | | | | |

## **Table S2. Institutional practices of public health sector officials: Legal accountability**

| **Variable** | **Total (n=44)** | **Territorial health secretaries (n=17)** | **Health sector managers (n=20)** | **Other positions (n=7)** |
| --- | --- | --- | --- | --- |
| **Frequency of awareness of tutela actions^1^ filed against the entity for failure to uphold the right to health, n (%)** | | | | |
| Very frequently | 19 (43.2) | 8 (47.1) | 7 (35.0) | 4 (57.1) |
| Sometimes | 15 (34.1) | 4 (23.5) | 9 (45.0) | 2 (28.6) |
| Never | 8 (18.2) | 5 (29.4) | 2 (10.0) | 1 (14.3) |
| Doesn't know | 2 (4.5) | 0 (0.0) | 2 (10.0) | 0 (0.0) |
| Note: n = number of participants; % = percentage within each category.  ^1^The tutela is a mechanism for protecting citizens' constitutional rights through judicial authorities when those rights are violated or threatened by the action or omission of public authorities [(1)](https://www.zotero.org/google-docs/?eSbYgk). | | | | |

## **Table S3 Decision-making dynamics of public health sector officials: Influence of external pressure on decision-making**

| **Variable** | **Total (n=44)** | **Territorial health secretaries (n=17)** | **Health sector managers (n=20)** | **Other positions (n=7)** |
| --- | --- | --- | --- | --- |
| **Perception of pressure from external groups or coalitions on public officials to intervene in health sector planning, n (%)** | | | | |
| Very pressured | 6 (13.6) | 3 (17.7) | 2 (10.0) | 1 (14.3) |
| Somewhat pressured | 19 (43.2) | 7 (41.2) | 9 (45.0) | 3 (42.9) |
| Not at all pressured | 8 (18.2) | 2 (11.8) | 4 (20.0) | 2 (28.6) |
| Doesn't know | 11 (25.0) | 5 (29.4) | 5 (25.0) | 1 (14.3) |
| **Possible consequences of pressure from external groups or coalitions on healthcare system planning, n (%)^1^** | | | | |
| Increased demand for services such as medical consultations and laboratory tests for patients | 9 (36.0) | 4 (40.0) | 4 (36.4) | 1 (25.0) |
| Increase in administrative procedures for patients | 14 (56.0) | 6 (60.0) | 6 (54.6) | 2 (50.0) |
| Increase in waiting times and problem resolution for patients | 18 (72.0) | 8 (80.0) | 7 (63.6) | 3 (75.0) |
| Increase in out-of-pocket expenses for patients | 11 (44.0) | 4 (40.0) | 5 (45.5) | 2 (50.0) |
| Increase in negative health outcomes for patients | 8 (32.0) | 2 (20.0) | 3 (27.3) | 3 (75.0) |
| Increase in costs for healthcare service providers | 10 (40.0) | 4 (40.0) | 4 (36.4) | 2 (50.0) |
| Increase in costs for the payer of healthcare services | 7 (28.0) | 2 (20.0) | 5 (45.5) | 0 (0.0) |
| **Perception of pressure from external groups or coalitions on public officials to suggest, propose, or oppose changes in financing mechanisms and budget allocation within the health sector, n (%)** | | | | |
| Very pressured | 4 (9.1) | 2 (11.8) | 1 (5.0) | 1 (14.3) |
| Somewhat pressured | 11 (25.0) | 5 (29.4) | 5 (25.0) | 1 (14.3) |
| Not at all pressured | 15 (34.1) | 4 (23.5) | 7 (35.0) | 4 (57.1) |
| Doesn't know | 14 (31.8) | 6 (35.3) | 7 (35.0) | 1 (14.3) |
| **Possible consequences of pressure from external groups or coalitions on financing mechanisms and budget planning in the health sector, n (%)^2^** | | | | |
| Increase in demand for services such as medical consultations and laboratory tests for patients | 8 (53.3) | 3 (42.9) | 5 (83.3) | 0 (0.0) |
| Increase in costs for healthcare service providers | 7 (46.7) | 4 (57.1) | 3 (50.0) | 0 (0.0) |
| Increase in costs for the payer of healthcare services | 7 (46.7) | 4 (57.1) | 3 (50.0) | 0 (0.0) |
| Increase in administrative procedures for patients | 11 (73.3) | 5 (71.4) | 4 (66.7) | 2 (100.0) |
| Increase in waiting times and problem resolution for patients | 8 (53.3) | 3 (42.9) | 4 (66.7) | 1 (50.0) |
| Increase in out-of-pocket expenses for patients | 8 (53.3) | 4 (57.1) | 3 (50.0) | 1 (50.0) |
| ^1^People who answered ‘very pressured’ or ‘somewhat pressured’ to the item on the perception of pressure from external groups or coalitions on public officials to intervene in health sector planning. ^2^People who answered ‘very pressured’ or ‘somewhat pressured’ to the item on the perception of pressure from external groups or coalitions on public officials to intervene in financing mechanisms and budget planning within the health sector.  Note: n = number of participants; % = percentage within each category. | | | | |

## **Table S4. Decision-making dynamics of public health sector officials: Influence on decision-making in the health sector**

| **Variable** | **Total (n=44)** | **Territorial health secretaries (n=17)** | **Health sector managers (n=20)** | **Other positions (n=7)** |
| --- | --- | --- | --- | --- |
| **Perception of need for greater decision-making power over the functioning of public hospitals and insurance programs in their territory, n (%)** | 34 (77.3) | 13 (76.5) | 14 (70.0) | 7 (100.0) |
| **Perception related to national government guidelines/regulations on how the health budget should be spent** | | | | |
| It is not possible to make adjustments between spending categories | 15 (34.1) | 4 (23.5) | 8 (40.0) | 3 (42.9) |
| It is possible in some cases to make adjustments between spending categories | 16 (36.4) | 7 (41.2) | 6 (30.0) | 3 (42.9) |
| It is always possible to make adjustments between spending categories | 15 (34.1) | 6 (35.3) | 7 (35.0) | 2 (28.6) |
| **Topics in which the actor has made decisions regarding financial allocations or expenditures, n (%)** | | | | |
| Enrollment of new beneficiaries | 6 (13.6) | 3 (17.7) | 1 (5.0) | 2 (28.6) |
| Payment to existing service providers | 3 (6.8) | 1 (5.9) | 2 (10.0) | 0 (0.0) |
| Contracting of new service providers | 3 (6.8) | 2 (11.8) | 1 (5.0) | 0 (0.0) |
| Essential capital expenditure (infrastructure, technology) | 7 (15.9) | 2 (11.8) | 4 (20.0) | 1 (14.3) |
| Flexible/discretionary spending | 1 (2.3) | 0 (0.0) | 1 (5.0) | 0 (0.0) |
| Financial support for public hospitals | 2 (4.5) | 0 (0.0) | 2 (10.0) | 0 (0.0) |
| Hiring of support and healthcare personnel to strengthen health processes | 3 (6.8) | 3 (17.7) | 0 (0.0) | 0 (0.0) |
| **Actions taken when disagreeing with directives/orders from national health agencies, authorities, or ministries during routine service delivery activities, n (%)** | | | | |
| I can only implement what they require | 23 (52.3) | 6 (35.3) | 12 (60.0) | 5 (71.4) |
| I go to the district administrator/mayor/chief district officer, who might take the case to the project directors/coordinators. | 12 (27.3) | 6 (35.3) | 5 (25.0) | 1 (14.3) |
| I write to the project directors/coordinators at the regional/national level to request their approval for changes. | 7 (15.9) | 3 (17.7) | 3 (15.0) | 1 (14.3) |
| Sometimes I only implement part of what they require. | 2 (4.6) | 2 (11.8) | 0 (0.0) | 0 (0.0) |
| **Suggested, recommended, or carried out the following action: Enroll new beneficiaries in programs, n (%)** | | | | |
| Suggested and successful | 20 (45.5) | 9 (52.9) | 9 (45.0) | 2 (28.6) |
| Suggested but unsuccessful | 8 (18.2) | 3 (17.7) | 4 (20.0) | 1 (14.3) |
| Not suggested | 16 (36.4) | 5 (29.4) | 7 (35.0) | 4 (57.1) |
| **Suggested, recommended, or carried out the following action: Start new clinics or medical offices, n (%)** | | | | |
| Suggested and successful | 8 (18.2) | 3 (17.7) | 4 (20.0) | 1 (14.3) |
| Suggested but unsuccessful | 8 (18.2) | 5 (29.4) | 2 (10.0) | 1 (14.3) |
| Not suggested | 28 (63.6) | 9 (52.9) | 14 (70.0) | 5 (71.4) |
| **Suggested, recommended, or carried out the following action: Extend staff/facility working hours, n (%)** | | | | |
| Suggested and successful | 5 (11.4) | 1 (5.9) | 4 (20.0) | 0 (0.0) |
| Suggested but unsuccessful | 8 (18.2) | 0 (0.0) | 6 (30.0) | 2 (28.6) |
| Not suggested | 31 (70.5) | 16 (94.1) | 10 (50.0) | 5 (71.4) |
| **Suggested, recommended, or carried out the following action: Introduce/improve complaint and grievance resolution mechanisms, n (%)** | | | | |
| Suggested and successful | 19 (43.2) | 9 (52.9) | 9 (45.0) | 1 (14.3) |
| Suggested but unsuccessful | 15 (34.1) | 8 (47.1) | 4 (20.0) | 3 (42.9) |
| Not suggested | 10 (22.7) | 0 (0.0) | 7 (35.0) | 3 (42.9) |
| **Suggested, recommended, or carried out the following action: Introduce new services (included in benefits packages), n (%)** | | | | |
| Suggested and successful | 11 (25.0) | 6 (35.3) | 4 (20.0) | 1 (14.3) |
| Suggested but unsuccessful | 8 (18.2) | 1 (5.9) | 6 (30.0) | 1 (14.3) |
| Not suggested | 25 (56.8) | 10 (58.8) | 10 (50.0) | 5 (71.4) |
| Note: n = number of participants; % = percentage within each category. | | | | |

## **Table S5. Perceptions of public health sector officials regarding the health system: Integration and coordination of the health system**

| **Variable** | **Total**  **(n=44)** | **Territorial health secretaries (n=17)** | **Health sector managers (n=20)** | **Other positions (n=7)** |
| --- | --- | --- | --- | --- |
| **Level of integration between health sector governing institutions and healthcare service provider institutions, n (%)** | | | | |
| Very integrated | 4 (9.1) | 1 (5.9) | 3 (15.0) | 0 (0.0) |
| Integrated | 14 (31.8) | 6 (35.3) | 4 (20.0) | 4 (57.1) |
| Moderately integrated | 18 (40.9) | 6 (35.3) | 9 (45.0) | 3 (42.9) |
| Slightly integrated | 8 (18.2) | 4 (23.5) | 4 (20.0) | 0 (0.0) |
| **Perception of integration between public and private sector information systems for the provision of healthcare services, n (%)** | | | | |
| A lot | 4 (9.1) | 1 (5.9) | 2 (10.0) | 1 (14.3) |
| Somewhat | 20 (45.5) | 8 (47.1) | 11 (55.0) | 1 (14.3) |
| Nothing | 20 (45.5) | 8 (47.1) | 7 (35.0) | 5 (71.4) |
| **Possible consequences of the lack of integrated information among providers, payers, and patients, n (%)** | | | | |
| Increase in medical tests for patients | 9 (20.5) | 3 (17.7) | 6 (30.0) | 0 (0.0) |
| Increase in administrative procedures for patients | 31 (70.5) | 10 (58.8) | 15 (75.0) | 6 (85.7) |
| Increase in waiting times and problem resolution for patients | 34 (77.3) | 12 (70.6) | 16 (80.0) | 6 (85.7) |
| Increase in out-of-pocket expenses for patients | 25 (56.8) | 7 (41.2) | 15 (75.0) | 3 (42.9) |
| Increase in negative health outcomes for patients | 21 (47.7) | 5 (29.4) | 12 (60.0) | 4 (57.1) |
| Increase in costs for healthcare service providers | 16 (36.4) | 8 (47.1) | 7 (35.0) | 1 (14.3) |
| Increase in costs for the payer of healthcare services | 15 (34.1) | 5 (29.4) | 9 (45.0) | 1 (14.3) |
| Note: n = number of participants; % = percentage within each category. | | | | |

## **Table S6. Perceptions of public health sector officials regarding the health system: Perceptions of health services and benefits**

| **Variable** | **Total**  **(n=44)** | **Territorial health secretaries (n=17)** | **Health sector managers (n=20)** | **Other positions (n=7)** |
| --- | --- | --- | --- | --- |
| **Perceived sufficiency of services and procedures included in the benefits plan based on the population’s needs, n (%)** | 12 (27.3) | 4 (23.5) | 7 (35.0) | 1 (14.3) |
| **Perception of sufficiency of the capitation payment unit (UPC) for managing health services and administrative costs in Colombia, n (%)** | | | | |
| Fully sufficient | 1 (2.3) | 1 (5.9) | 0 (0.0) | 0 (0.0) |
| Sufficient | 7 (15.9) | 2 (11.8) | 3 (15.0) | 2 (28.6) |
| Insufficient | 30 (68.2) | 14 (82.4) | 11 (55.0) | 5 (71.4) |
| Totally insufficient | 6 (13.6) | 0 (0.0) | 6 (30.0) | 0 (0.0) |
| Note: n = number of participants; % = percentage within each category. | | | | |

## **Table S7. Perceptions of public health sector officials regarding the health system: Financing mechanisms and payment methods**

| **Variable** | **Total**  **(n=44)** | **Territorial health secretaries (n=17)** | **Health sector managers (n=20)** | **Other positions (n=7)** |
| --- | --- | --- | --- | --- |
| **Perception of timely payment to healthcare service providers, n (%)** | | | | |
| Always | 2 (4.6) | 1 (5.9) | 1 (5.0) | 0 (0.0) |
| Almost always | 8 (18.2) | 2 (11.8) | 5 (25.0) | 1 (14.3) |
| Sometimes | 24 (54.6) | 9 (52.9) | 11 (55.0) | 4 (57.1) |
| Never | 10 (22.7) | 5 (29.4) | 3 (15.0) | 2 (28.6) |
| **Perception of differential rates among healthcare service providers for the same services, n (%)** | | | | |
| For no services | 3 (6.8) | 2 (11.8) | 1 (5.0) | 0 (0.0) |
| For some services | 38 (86.4) | 15 (88.2) | 16 (80.0) | 7 (100.0) |
| For all services | 3 (6.8) | 0 (0.0) | 3 (15.0) | 0 (0.0) |
| Note: n = number of participants; % = percentage within each category. | | | | |

## **Table S8. Perceptions of public health sector officials regarding the health system: Participation of state officials with the community**

| **Variable** | **Total**  **(n=44)** | **Territorial health secretaries (n=17)** | **Health sector managers (n=20)** | **Other positions (n=7)** |
| --- | --- | --- | --- | --- |
| **Participation of state officials in their interaction with the community on public health matters, n (%)** | | | | |
| Unsatisfactory | 4 (9.1) | 1 (5.9) | 1 (5.0) | 2 (28.6) |
| Partially satisfactory | 26 (59.1) | 12 (70.6) | 12 (60.0) | 2 (28.6) |
| Satisfactory | 14 (31.8) | 4 (23.5) | 7 (35.0) | 3 (42.9) |
| Note: n = number of participants; % = percentage within each category. | | | | |

## **Table S9. Perceptions of public health sector officials regarding the health system: Health system surveillance and control**

| **Variable** | **Total**  **(n=44)** | **Territorial health secretaries (n=17)** | **Health sector managers (n=20)** | **Other positions (n=7)** |
| --- | --- | --- | --- | --- |
| **Perceived effectiveness of the National Superintendency of Health in overseeing, n (%)** | | | | |
| Very effective | 8 (18.2) | 4 (23.5) | 3 (15.0) | 1 (14.3) |
| Somewhat effective | 23 (52.3) | 9 (53.0) | 11 (55.0) | 3 (42.9) |
| Not effective at all | 13 (29.6) | 4 (23.5) | 6 (30.0) | 3 (42.9) |
| **Perceived effectiveness of the Office of the Comptroller in overseeing and regulating the health system in Colombia, n (%)** | | | | |
| Very effective | 13 (29.6) | 7 (41.2) | 5 (25.0) | 1 (14.3) |
| Somewhat effective | 22 (50.0) | 6 (35.3) | 12 (60.0) | 4 (57.1) |
| Not effective at all | 8 (18.2) | 3 (17.7) | 3 (15.0) | 2 (28.6) |
| Does not know | 1 (2.3) | 1 (5.9) | 0 (0.0) | 0 (0.0) |
| **Perceived effectiveness of the Office of the Inspector General in overseeing and regulating the health system in Colombia, n (%)** | | | | |
| Very effective | 13 (29.6) | 7 (41.2) | 5 (25.0) | 1 (14.3) |
| Somewhat effective | 24 (54.6) | 7 (41.2) | 12 (60.0) | 5 (71.4) |
| Not effective at all | 6 (13.6) | 2 (11.8) | 3 (15.0) | 1 (14.3) |
| Doesn't know | 1 (2.3) | 1 (5.9) | 0 (0.0) | 0 (0.0) |
| Note: n = number of participants; % = percentage within each category. | | | | |

# **References**

[1.](https://www.zotero.org/google-docs/?UvkbZE) [Gobierno de Colombia. Constitución Política De Colombia [Internet]. 1991. Available from: https://colombia.justia.com/nacionales/constitucion-politica-de-colombia/](https://www.zotero.org/google-docs/?UvkbZE)
